# Supplementary material for: Tunable molecular separation by nanoporous membranes
Source: Nat Commun. 2016 Dec 20;7:13872. doi: 10.1038/ncomms13872 (PMC5187437; doi:10.1038/ncomms13872)
Supplement: Supplementary Information — Supplementary Figures. [file ncomms13872-s1.pdf]

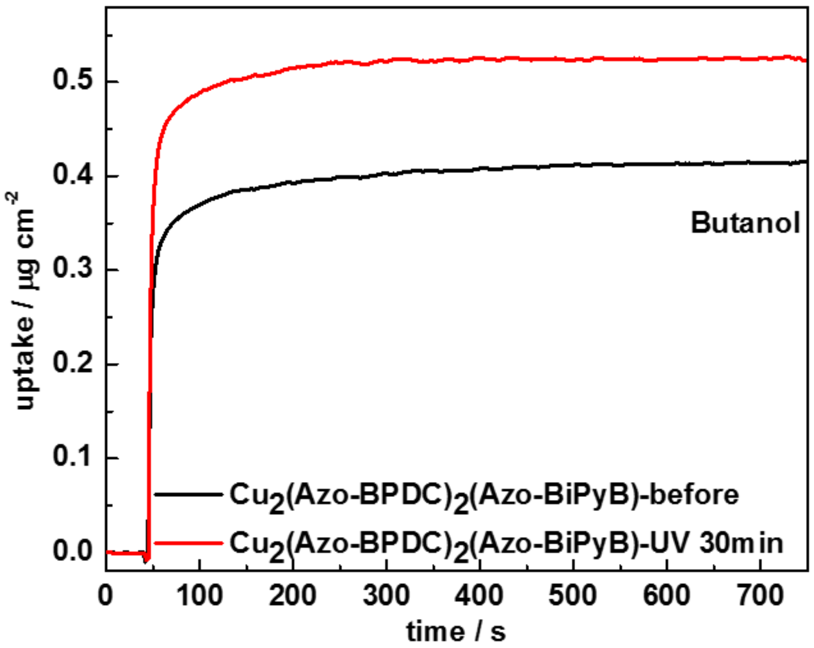

Supplementary Figure 1: The QCM uptake of butanol by the $Cu_2(AzoBPDC)_2(AzoBiPyB)$ SURMOF. The black line shows the uptake by the SURMOFs before UV irradiation and the red line shows the uptake by the same samples after UV irradiation for 30 min.

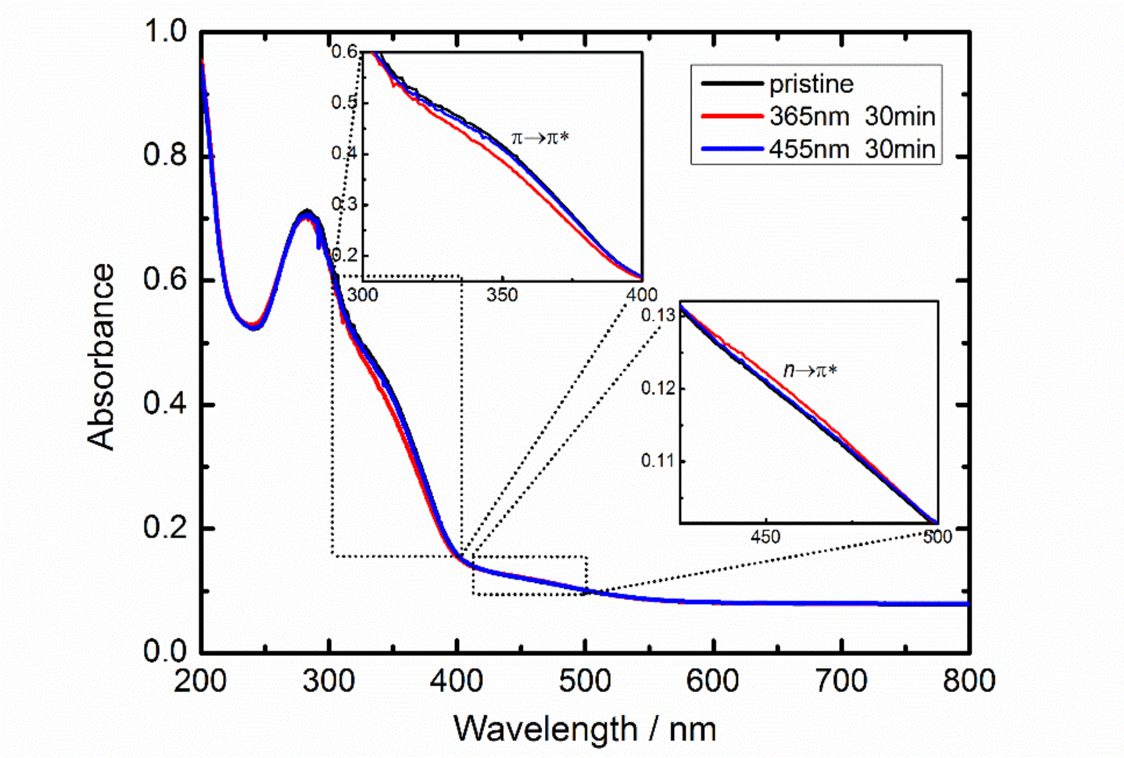

Supplementary Figure 2: UV-vis spectra of the $Cu_2(AzoBPDC)_2(AzoBiPyB)$ SURMOF on quartz glass. The spectra are measured in transmission mode. The large absorption band at about 280 nm is caused by the copper-paddle-wheel complex in the MOF (see, e.g., ref. DOI: 10.1063/1.4934737). A decrease of the π- π^* band and an increase of the n- π^* band can be observed upon UV irradiation. Irradiation with 455 nm results in virtually the pristine spectra.

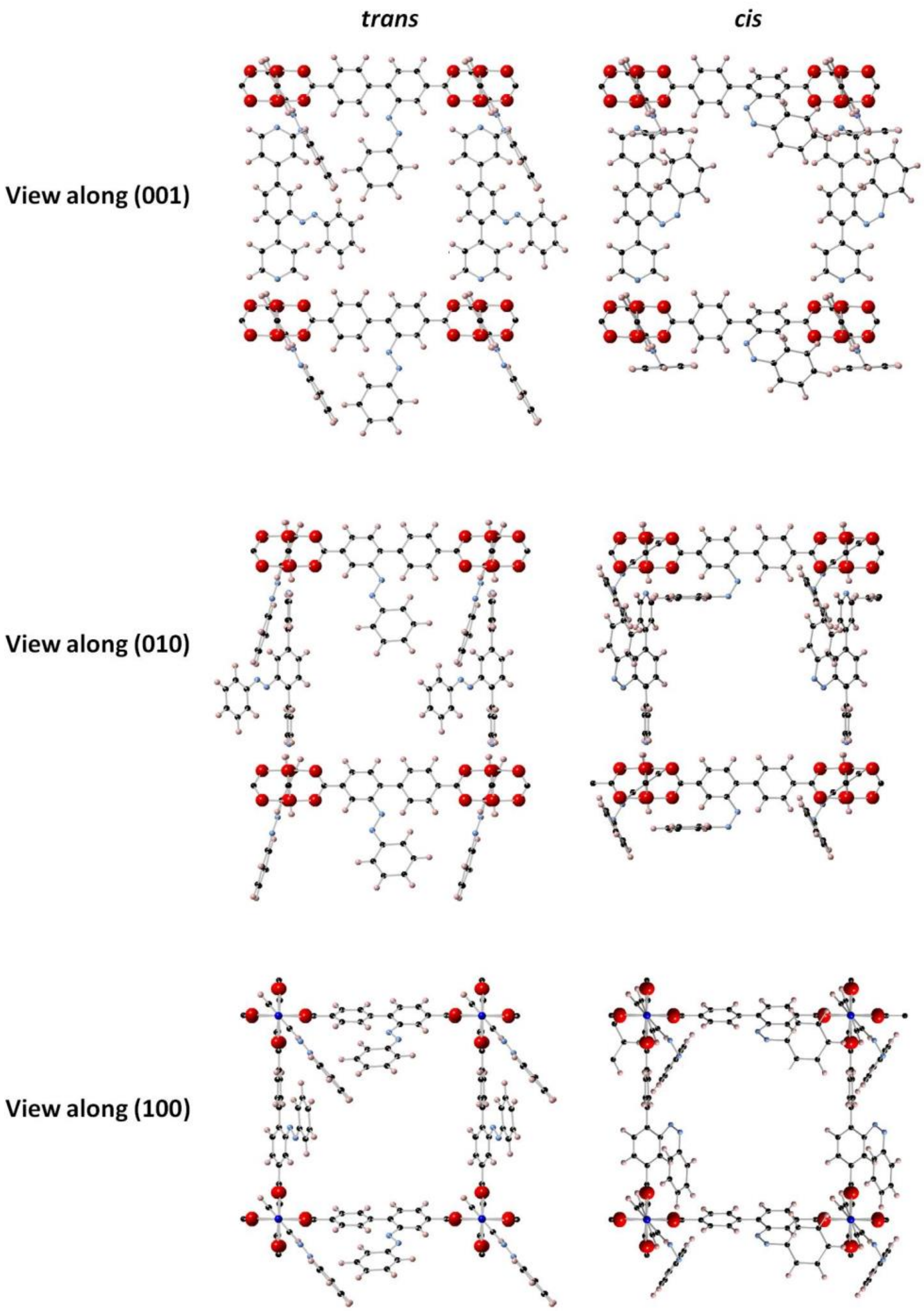

Supplementary Figure 3: Comparison of the $Cu_2(AzoBPDC)_2(AzoBiPyB)$ SURMOF structure with the azobenzene side groups in the *trans* and the *cis* state. It should be noted that the phenyl rings, and thus the azobenzene side groups, can rotate around the bond axis. Therefore, the positions of the azobenzene side groups are not fixed (or "frozen") as depicted here. For comparison, a 3D image of the structure from a different perspective is shown in Figure 2a.

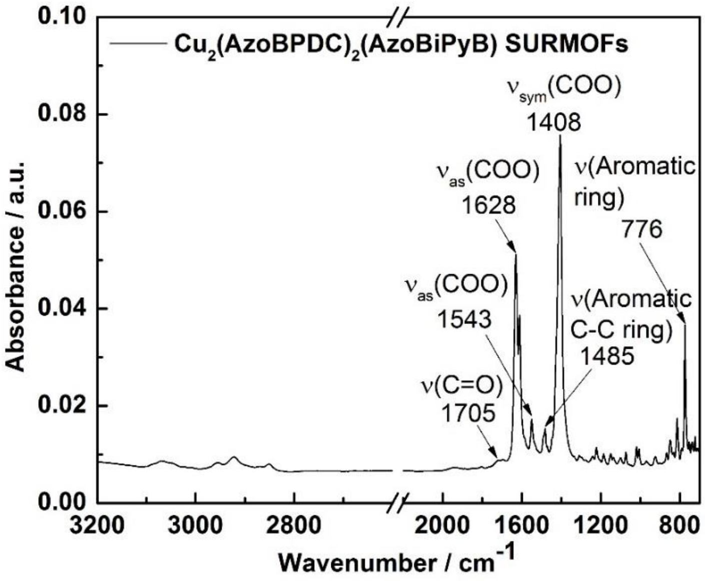

Supplementary Figure 4: IRRAS spectrum of $Cu_2(AzoBPDC)_2(AzoBiPyB)$ SURMOFs. The sample is grown on a MUD-SAM-modified gold substrate.

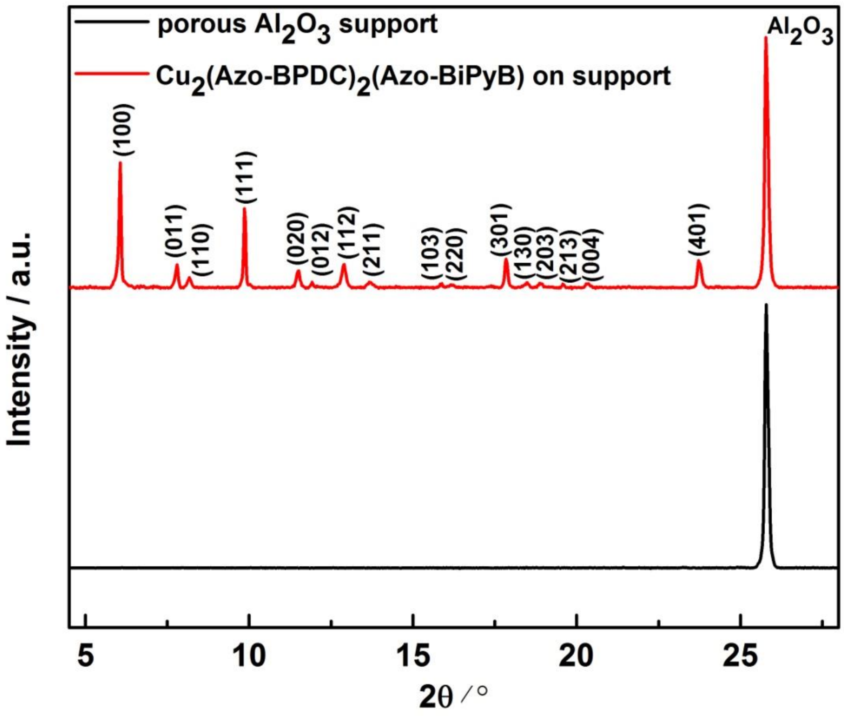

Supplementary Figure 5: X-ray diffractogram of the porous Al_2O_3 support (black) and the Cu_2(AzoBPDC)$_2$(AzoBiPyB) SURMOFs grown on top of the Al_2O_3 support (red).

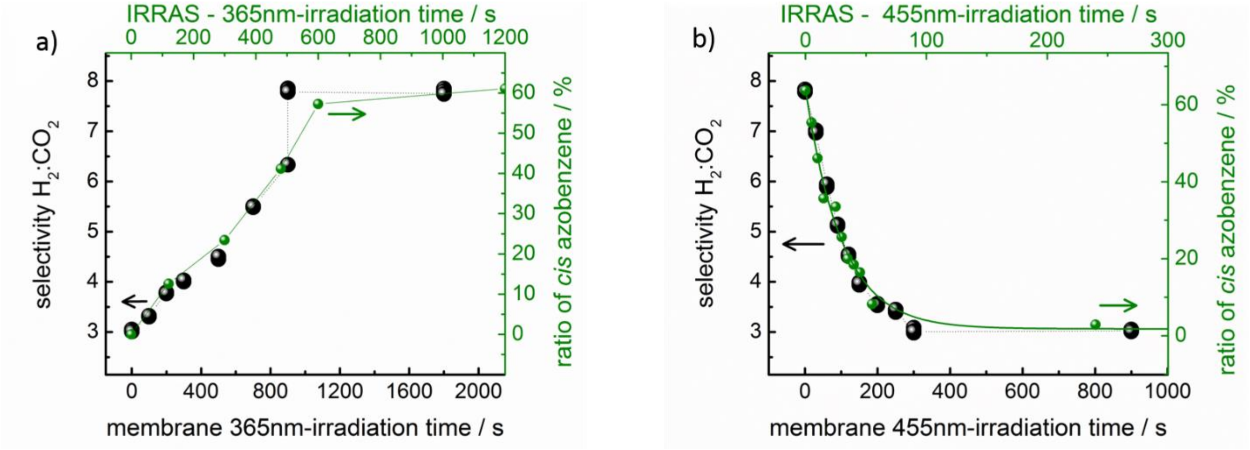

Supplementary Figure 6: Comparison of the membrane selectivity and the *cis* azobenzene ratio. The black sphere are the membrane data of Figure 6a) and b), while the small green spheres are the IRRAS data of *cis* azobenzene as shown in Figure 3a and 3b. Both data show coinciding characteristics, enabling the correlation shown in Figure 6c.
